# Supplementary material for: The Legionella collagen-like protein employs a unique binding mechanism for the recognition of host glycosaminoglycans
Source: bioRxiv. 2023 Dec 10:2023.12.10.570962. Preprint. [Version 1] doi: 10.1101/2023.12.10.570962 (PMC10723406; doi:10.1101/2023.12.10.570962)
Supplement: Supplement 1 [file media-1.pdf]

Supplementary Information

The *Legionella* collagen-like protein employs a unique binding mechanism for the recognition of host glycosaminoglycans

Saima Rehman[1], Anna K. Antonovic[2], Ian E. McIntire[3], Huaixin Zheng[3], Leanne Cleaver[1], Carlton O. Adams[3], Theo Portlock[1,4], Katherine Richardson[4], Rosie Shaw[4], Alain Oregioni[5], Giulia Mastroianni[2], Sara B-M. Whittaker[6], Geoff Kelly[5], Arianna Fornili[2*], Nicholas P. Cianciotto[3*] & James A. Garnett[1,4*]

[1]Centre for Host-Microbiome Interactions, Faculty of Dental, Oral & Craniofacial Sciences, King's College London, London, UK

[2]School of Physical and Chemical Sciences, Queen Mary University of London, London, UK

[3]Department of Microbiology and Immunology, Northwestern University Feinberg School of Medicine, Chicago, Illinois, USA

[4]School of Biological and Behavioural Sciences, Queen Mary University of London, London, UK

[5]The Medical Research Council Biomedical NMR Centre, the Francis Crick Institute, 1 Midland Road, London NW1 1AT, UK

[6]School of Cancer Sciences, College of Medical and Dental Sciences, University of Birmingham, Birmingham, UK

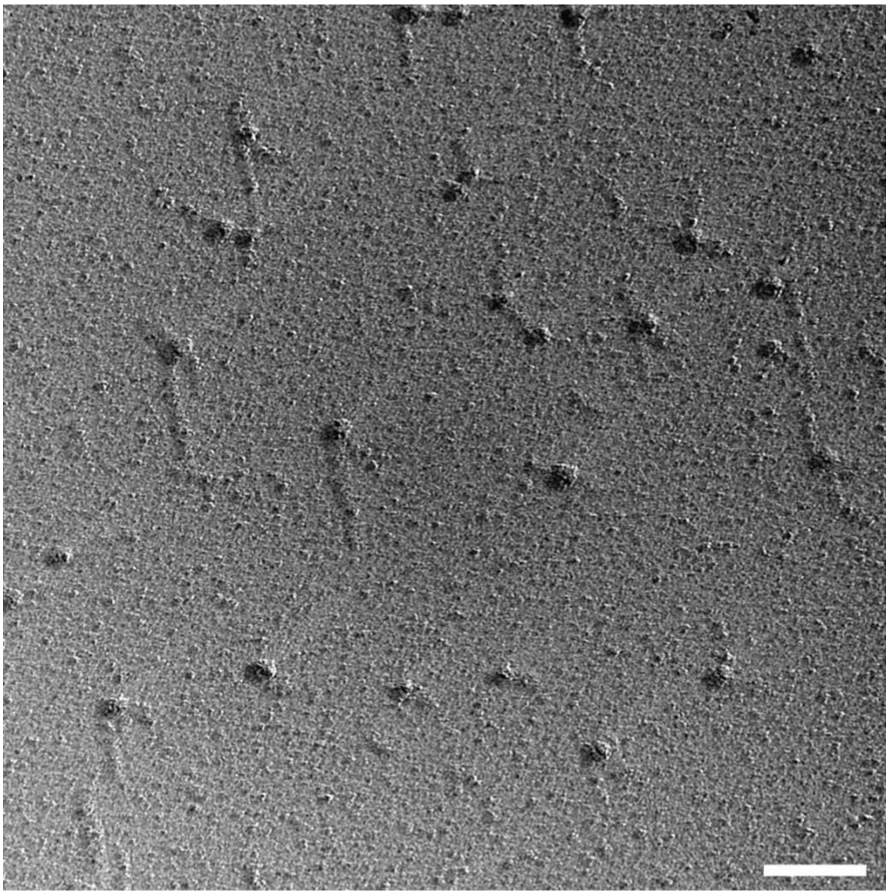

Supplementary Fig. 1 | Rotary shadowing electron microscopy of Lcl. Micrograph showing lollipop-shaped structures of Lcl trimers. The globular shapes correspond to trimeric C-terminal domains (CTD), while the stalks contain a trimeric collagen triple-helix (CLR). Some globular heads are missing the stalk region due to proteolysis. Variation in stalk lengths is due to differences in the platinum film thickness. The concentration of Lcl was 5 µg/ml. Scale bar: 50 nm.

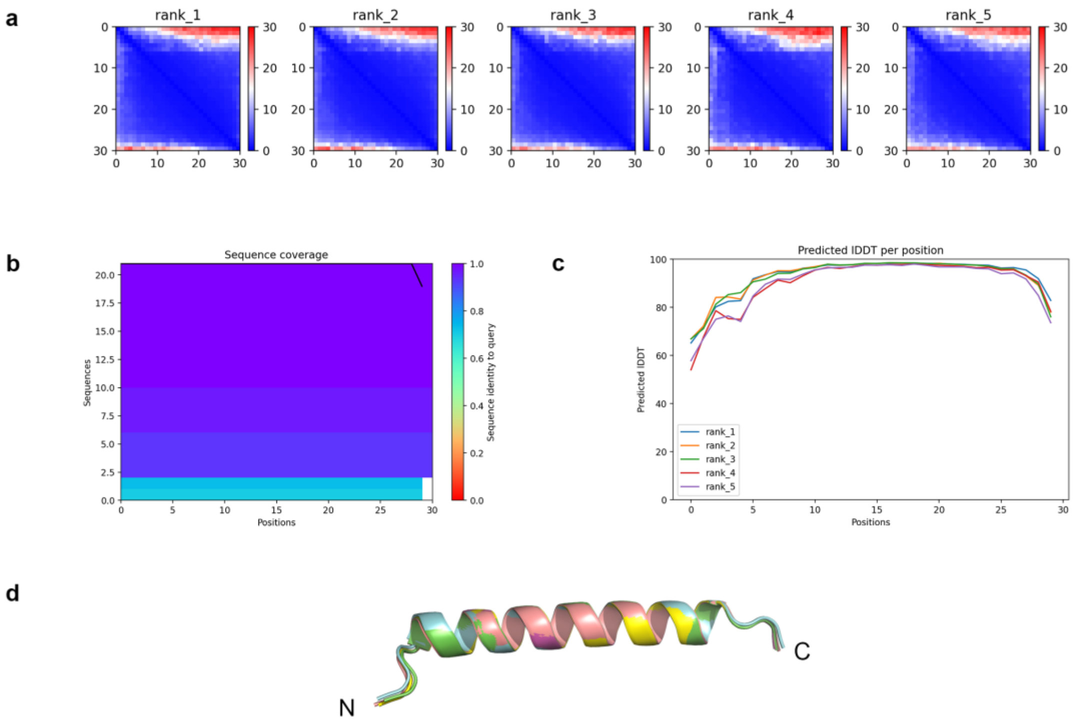

Supplementary Fig. 2 | AlphaFold2 modelling of Lcl-N. (a) Predicted Aligned Error (PAE) analysis of the five top ranking models. Low PAE values throughout the matrix indicates a well folded single domain. (b) Sequence coverage and identity used during modelling. (c) Local Distance Difference Test (lDDT) analysis against residue position for the five top ranking models. High lDDT values (<90) indicate a high accuracy in atomic positions for that residue. Lower values (<70) indicate low confidence atomic positioning, which is observed in dynamic regions. (d) Superposed top five ranked models with the termini highlighted (N/C: N-/C-terminus).

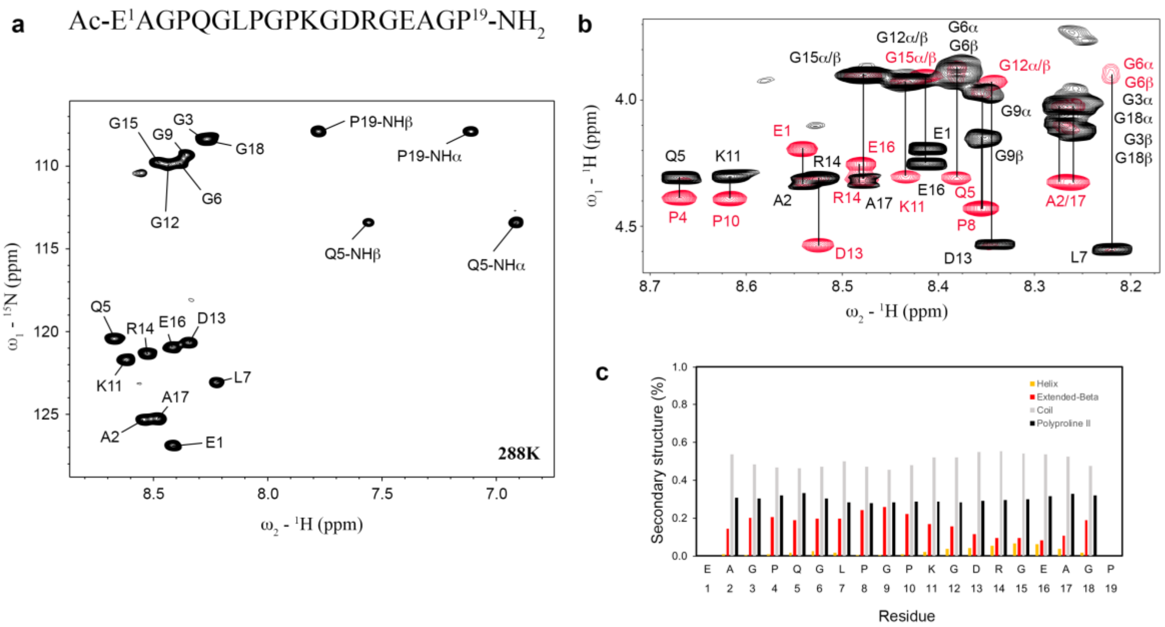

Supplementary Fig. 3 | Solution NMR spectroscopy analyses of unlabelled CLR peptide recorded at 15°C. a, ^{1}H–^{15}N SOFAST-HMQC spectrum of the CLR peptide with amide resonance assignments shown. Peptide sequence is shown above. **b,** ^{1}H–^{1}H TOCSY (black) and ^{1}H–^{1}H ROESY (red) spectra expanded on the NH-H_{α} region. A typical pattern of polyproline II structure is seen with strong NOE correlation observed between NH (i) and H_{α} (i-1). **c,** Secondary structure propensity of monomeric CLR peptide derived from backbone chemical shifts (C_{α}, C_{β}, H_{α}, N, NH) calculated using δ2D[1].

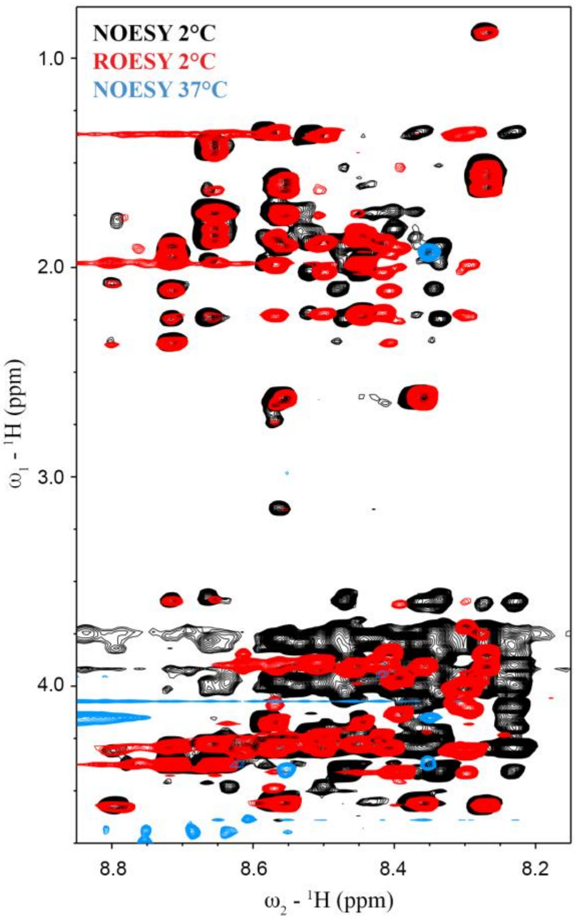

Supplementary Fig. 4 | Solution NMR spectroscopy analyses of $^{13}C^{15}N$ glycine labelled CLR peptide. ^{1}H–^{1}H NOESY spectra (mixing time 240 ms) recorded at 2°C (black) and 37°C (blue), and ^{1}H–^{1}H ROESY (mixing time 200 ms) spectrum recorded at 2°C (red) expanded on the amide region. Significant differences are observed between the NOESY and ROESY spectra at 2°C and significant spectral broadening is observed in the NOESY spectrum at 37°C. This indicates that the peptide is in equilibrium between monomeric and trimeric states.

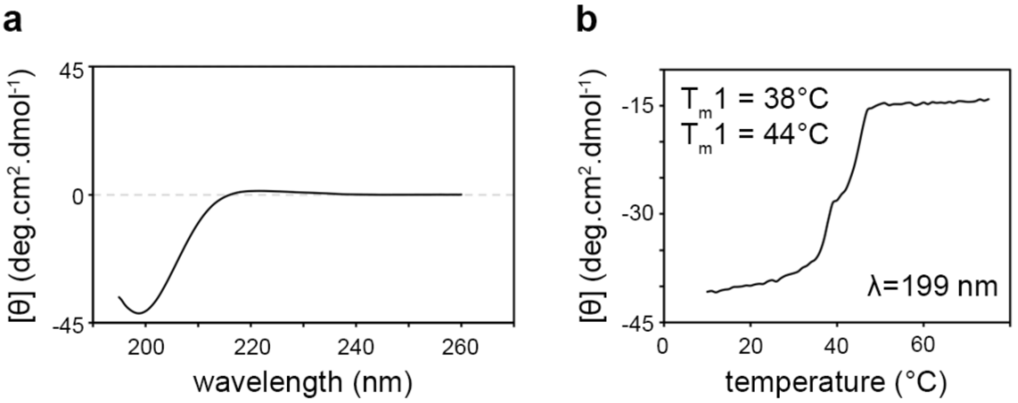

Supplementary Fig. 5 | Circular dichroism (CD) spectra of Lcl. a, CD spectra of Lcl recorded at 10°C with negative peak at 199 nm and maximum peak at 222 nm. **b,** Thermal denaturation of Lcl recorded between 10°C and 75°C at 199 nm. Two melting temperatures were determined at 38°C and 44°C.

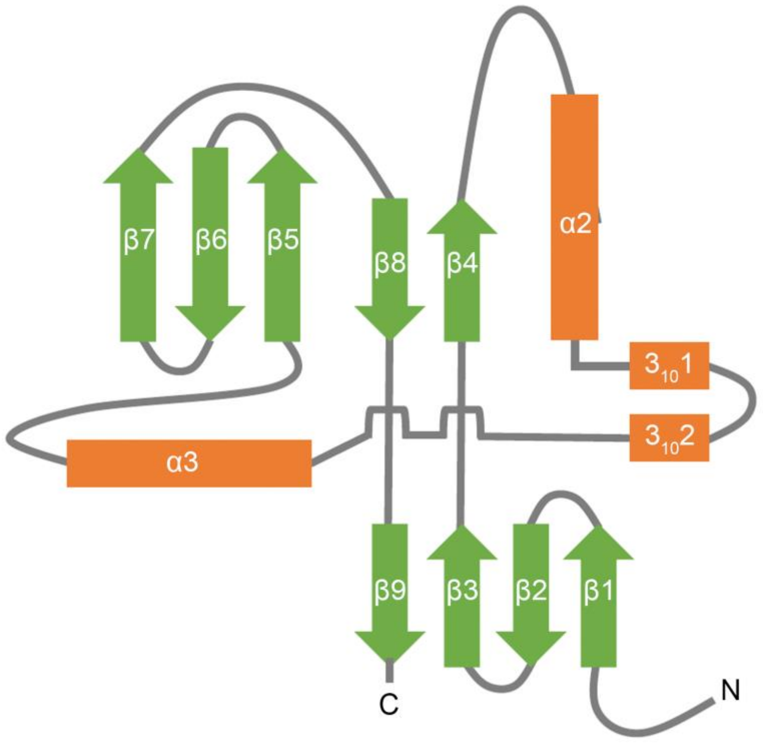

Supplementary Fig. 6 | Topology diagram for Lcl-CTD. Schematic representation of an individual Lcl-CTD domain. α-helix and 3_{10}-helix secondary structure are coloured orange, β-sheet secondary structure is coloured green, and loops are grey. Helices are labelled 1-2 and β-sheets are labelled 1-9.

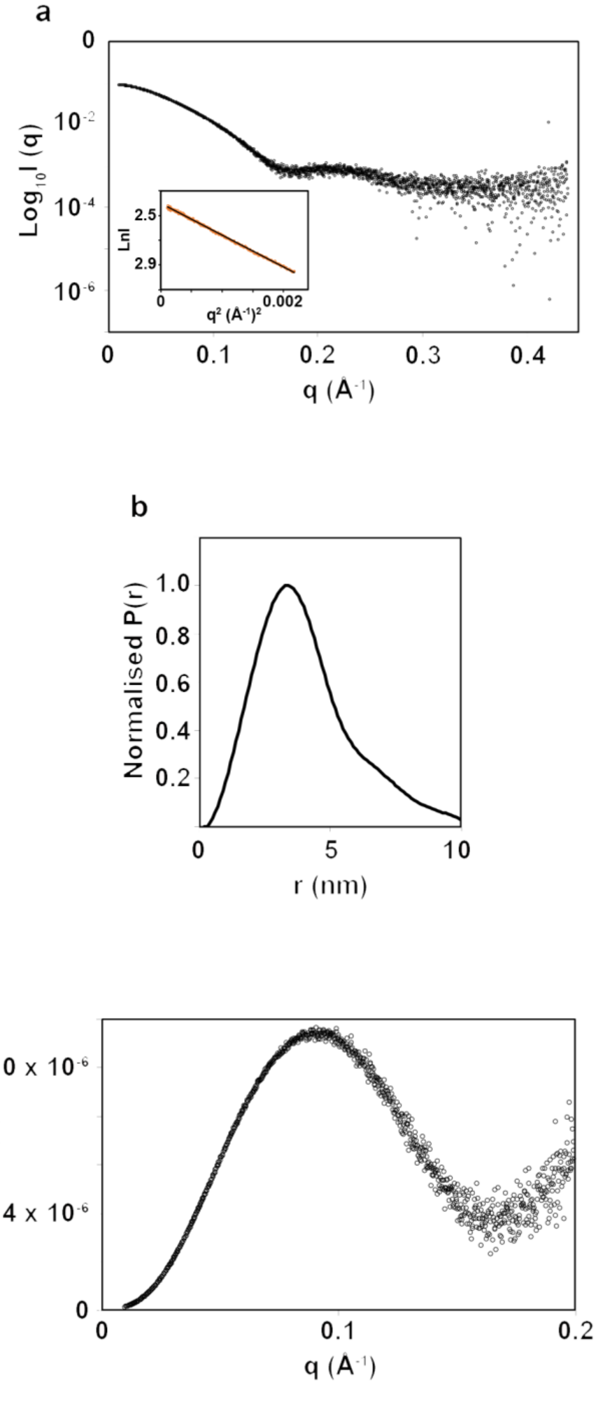

Supplementary Fig. 7 | SAXS analysis of Lcl-CTD. a, Experimental scattering curve of Lcl-CTD (black open circles). Inset: Guinier region (orange open circles) and linear regression (black line) for R_g evaluation. **b,** Shape distribution [P(r)] function derived from SAXS analysis for Lcl-CTD. **c,** Kratky, plot indicates that Lcl-CTD has dynamic properties in solution.

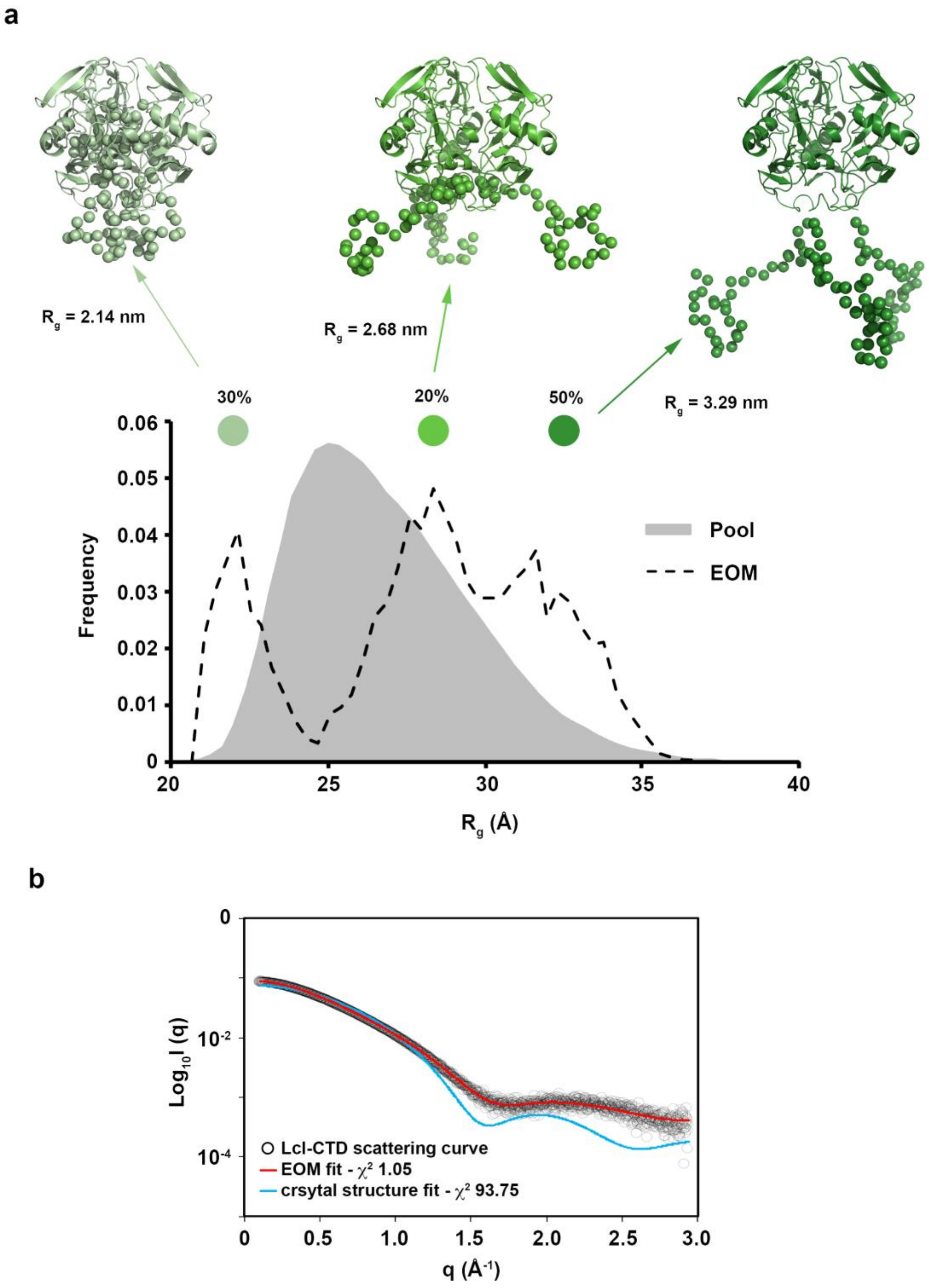

Supplementary Fig. 8 | SAXS derived model of Lcl-CTD in solution. a, EOM yielded three distinct populations: extended N-terminus (50%), partially extended N-terminus (20%) and compact N-terminus (30%). Lcl-CTD models corresponding to the centre of each population are shown. **b,** EOM (red line) and crystal structure (blue) fit to the Lcl-CTD SAXS data (black open circles) with χ^2 of 1.05 and 93.75, respectively.

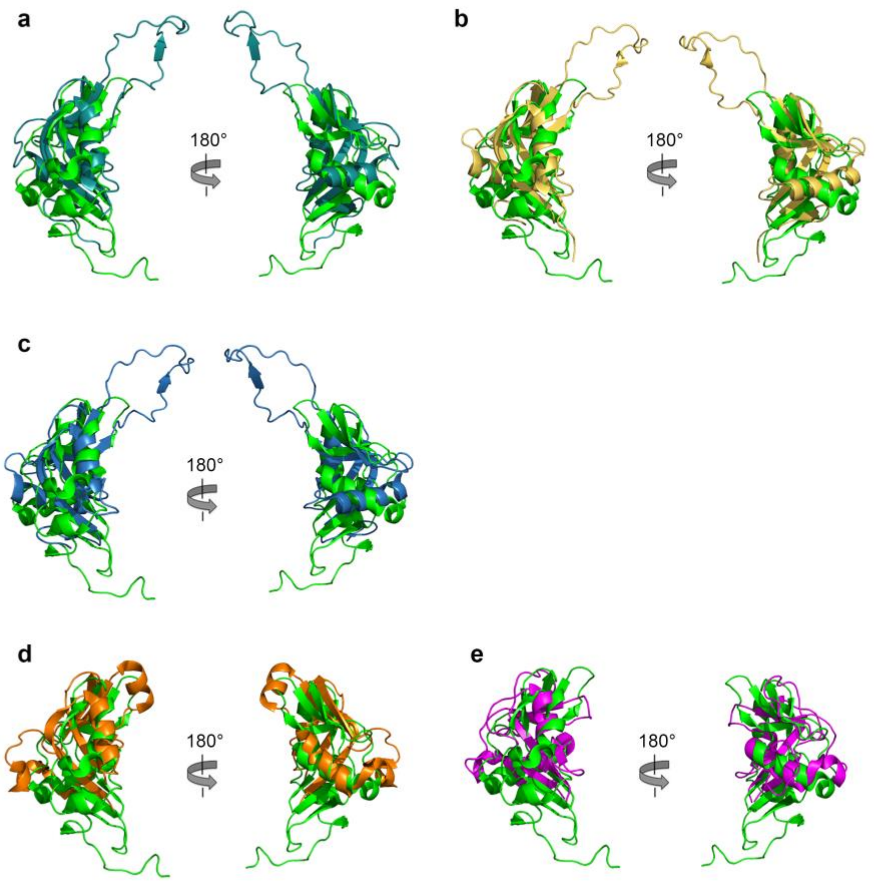

Supplementary Fig. 9 | Structural comparison of the Lcl-CTD monomer with other C-type lectin-like domains. Homologous structures identified using the Dali server with Z-scores >8.0 are shown[2]. **a,** Cartoon representation of Lcl-CTD (green) superimposed on the platelet glycoprotein Ib-α binding protein mucrocetin from the venom of *Trimeresurus mucrosquamatus* (teal; Protein Data Bank (PDB) ID code 1v4l[3]; chain B residues 201–325; Z-score 9.7; root mean squared deviated (rmsd) 2.7 Å). **b,** Cartoon representation of Lcl-CTD (green) superimposed on the integrin binding protein EMS16 from the venom of *Echis multisquamatus* (yellow; PDB ID code 1v7p[4]; chain A residues 1–127; Z-score 9.1; rmsd 2.7 Å). **c,** Cartoon representation of Lcl-CTD (green) superimposed on the integrin binding protein rhodocetin from the venom of *Calloselasma rhodostoma* (blue; PDB ID code 6nd8; chain B residues 1–122; Z-score 8.5; rmsd 2.8 Å). **d,** Cartoon representation of Lcl-CTD (green) superimposed on the adhesin protein intimin from enteropathogenic *Escherichia coli* (orange; PDB ID code 1f00[5]; chain I residues 840–939; Z-score 8.3; rmsd 3.5 Å). **e,** Cartoon representation of Lcl-CTD (green) superimposed on the integrin binding protein invasin from *Yersinia pseudotuberculosis* (purple; PDB ID code 1cwv[6]; chain A residues 887–986; Z-score 8.1; rmsd 2.9 Å).

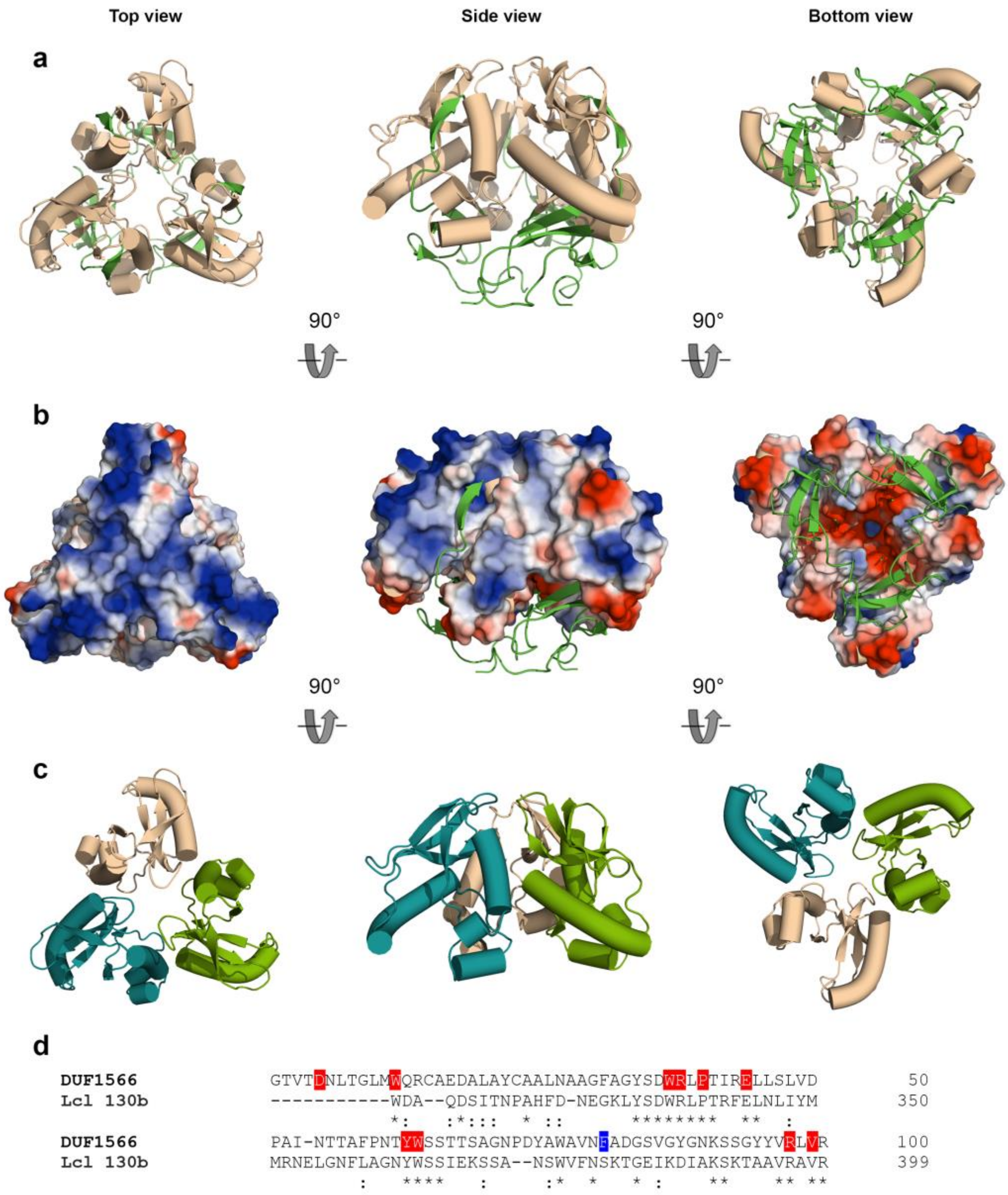

Supplementary Fig. 10 | DUF1566/pfam07603 motif within Lcl-CTD. a, Cartoon representation of trimeric Lcl-CTD with the DUF1566 regions coloured wheat, and the non-DUF1566 S1-S4 strands of Lcl-CTD coloured green. **b,** Electrostatic surface potential representation of the DUF1566 region within the Lcl-CTD trimer, with the Lcl-CTD S1-S4 strands shown as green cartoon. **c,** Cartoon representation of the isolated DUF1566 trimer from Lcl-CTD. **d,** Sequence alignment of the consensus DUF1566 motif and the corresponding region within *L. pneumophila* 130b Lcl. Identical residues are highlighted with an asterisk (*), and residues that are similar are highlighted with a colon (:). Completely conserved positions in the DUF1566 sequence that are also identical in Lcl are coloured red, and those that are not identical are coloured blue.

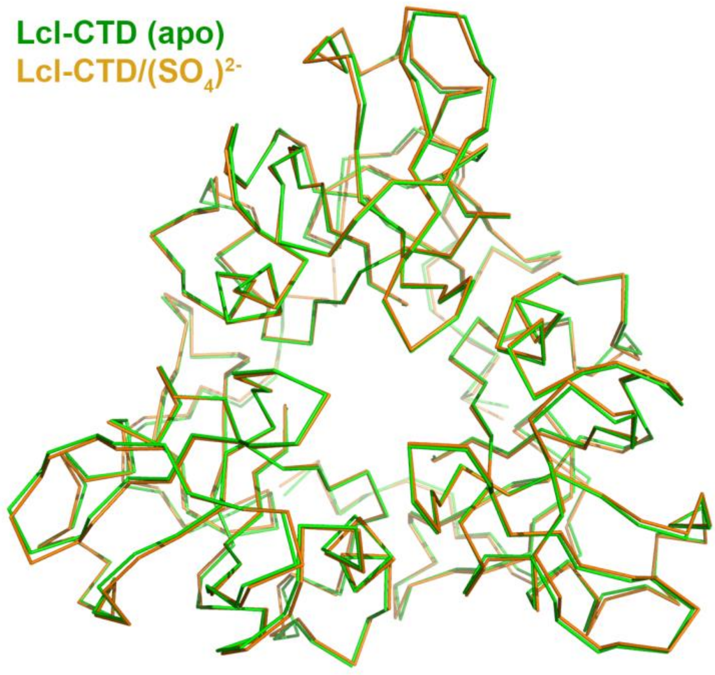

Supplementary Fig. 11 | Structural comparison of the Lcl-CTD trimer is the presence and absence of sulphate ions. Lcl-CTD trimer backbone structures are shown as ribbons in green (no sulphate) and orange (bound to sulphate ions).

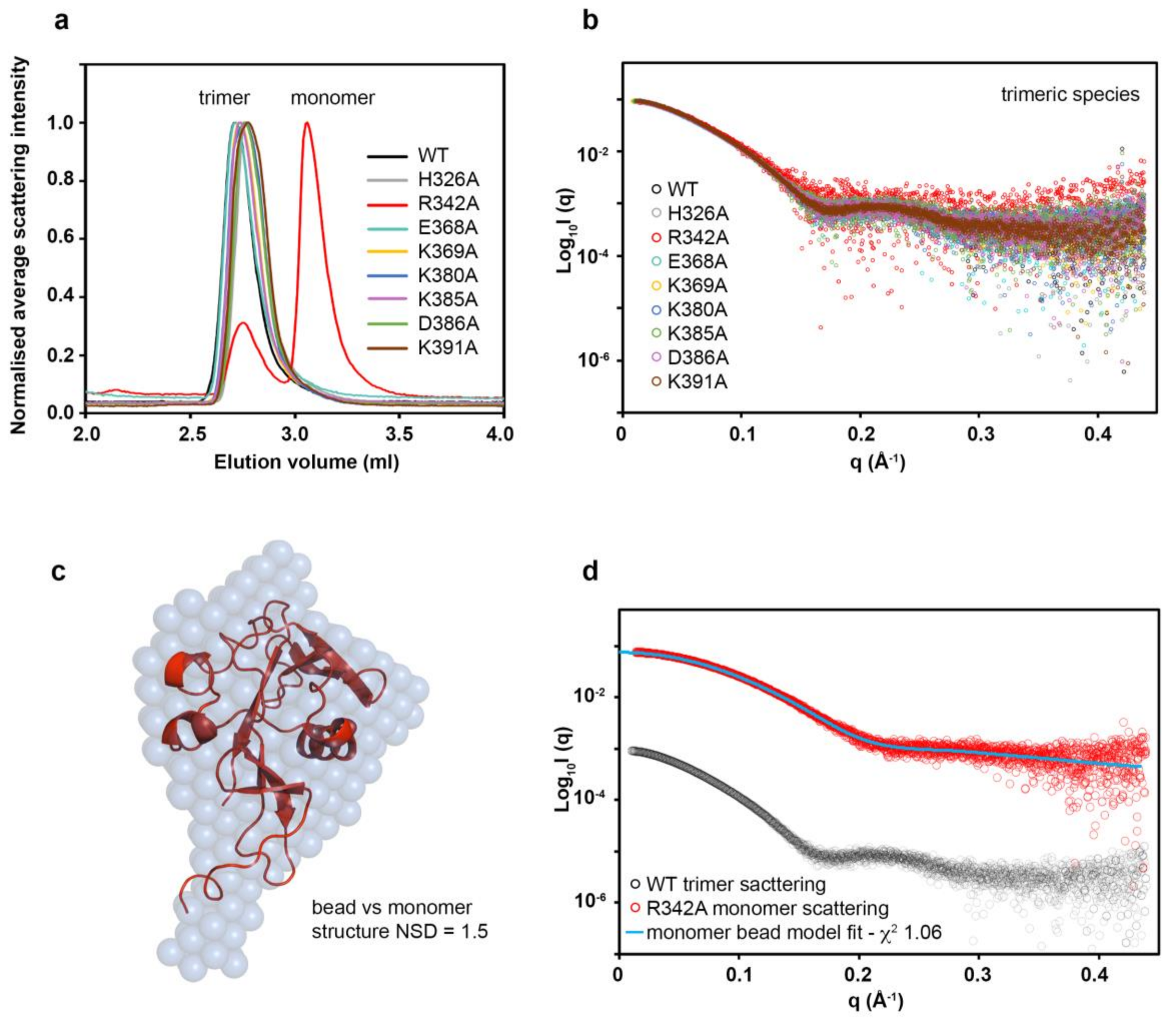

Supplementary Fig. 12 | SAXS analysis of Lcl-CTD mutants. a, Size-exclusion chromatography coupled with SAXS profile of wild-type and engineered Lcl-CTD. **b,** Experimental scattering curves of trimeric forms of wild-type and engineered Lcl-CTD. **c,** DAMMIF bead model of monomeric Lcl-CTD R342A superimposed with chain A from Lcl-CTD, with normalized spatial discrepancy (NSD) score of 1.5. **d,** Monomer Lcl-CTD crystal structure fit to monomeric Lcl-CTD R342A SAXS data (red open circles) with χ^2 of 1.06. Experimental scattering curve of trimeric wild-type Lcl-CTD (black open circles) is shown for comparison.

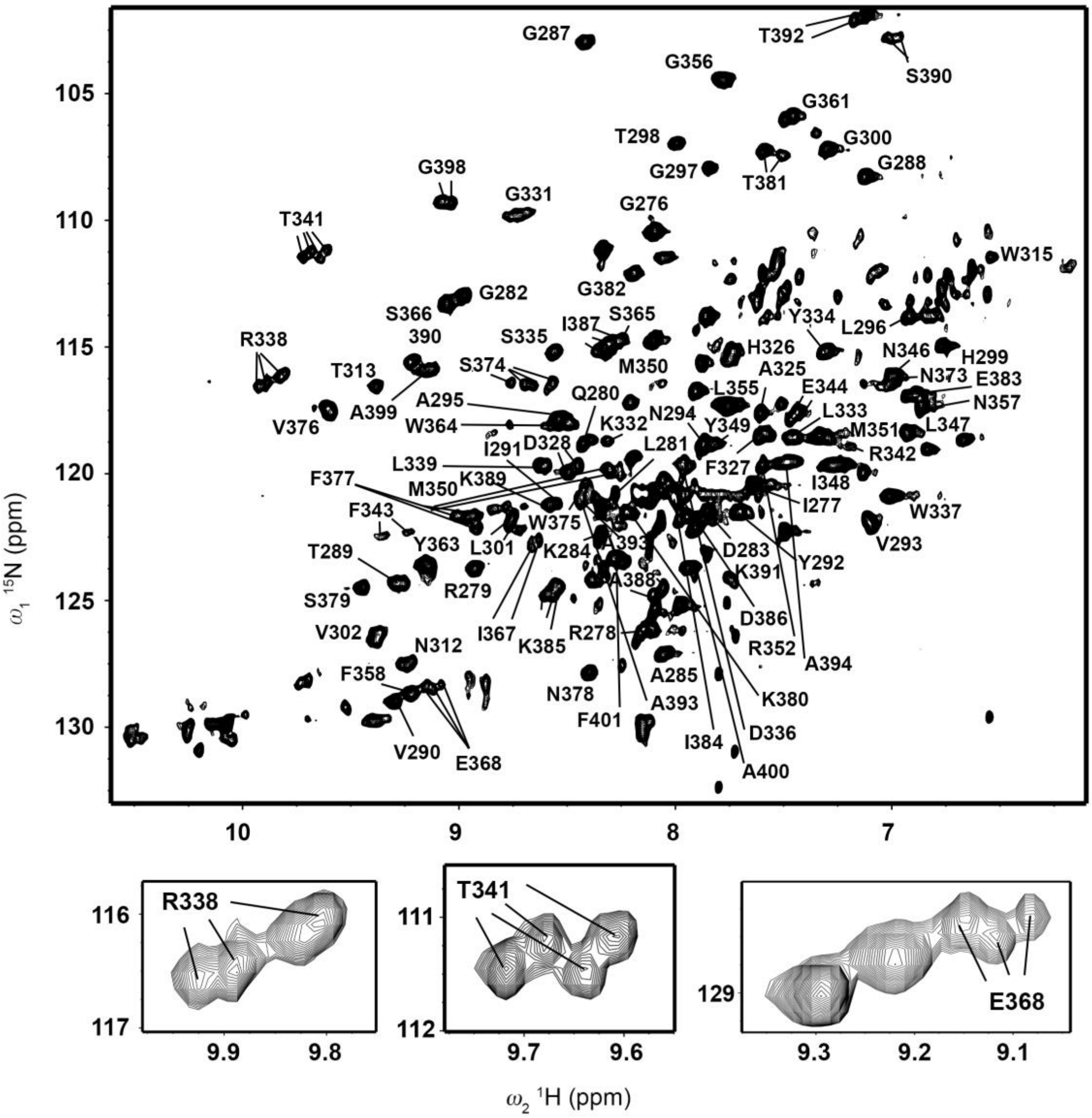

Supplementary Fig. 13 | Solution NMR spectroscopy of the Lcl C-terminal domain. ^{1}H–^{15}N HSQC TROSY spectrum with backbone amide resonance assignments shown. Boxed out areas are examples of residues that display conformation exchange.

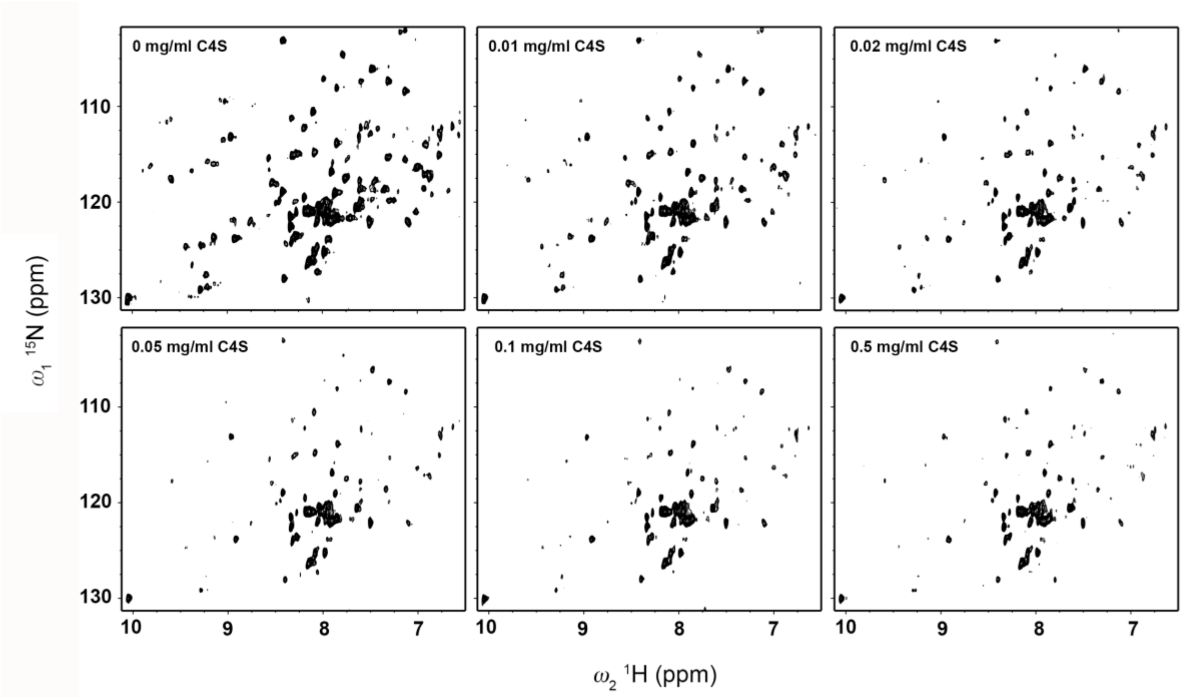

Supplementary Fig. 14 | NMR titration of C4S against Lcl-CTD. ^{1}H–^{15}N HSQC TROSY spectra of $^{2}H^{15}N$ labelled Lcl-CTD incubated with increasing concentrations of C4S extracted from bovine trachea.

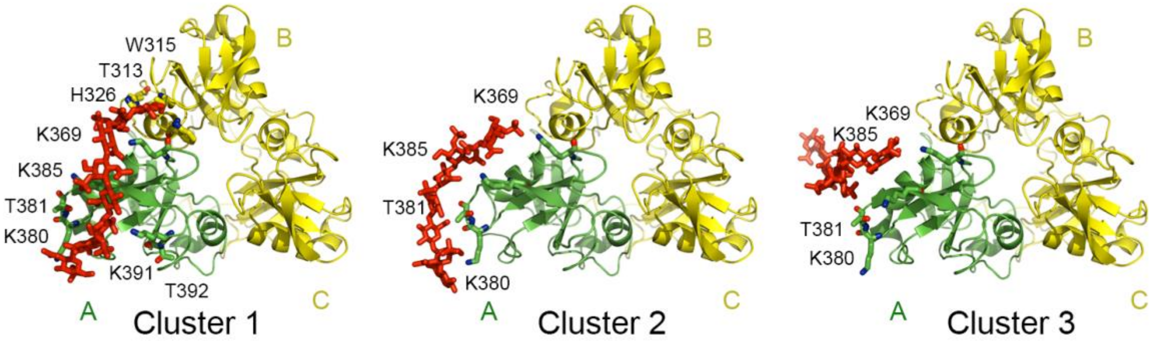

Supplementary Fig. 15 | Docking of C4S dp8 against monomeric Lcl-CTD. Top three HADDOCK clusters are shown. C4S is shown as sticks and coloured red, while monomeric Lcl-CTD is shown as a green cartoon. Docking was carried out on monomeric Lcl-CTD, but here it is shown superimposed onto the crystal trimer (yellow). The trimeric structure shown in Cluster 1 is equivalent to the HM model used for subsequent MD simulations.

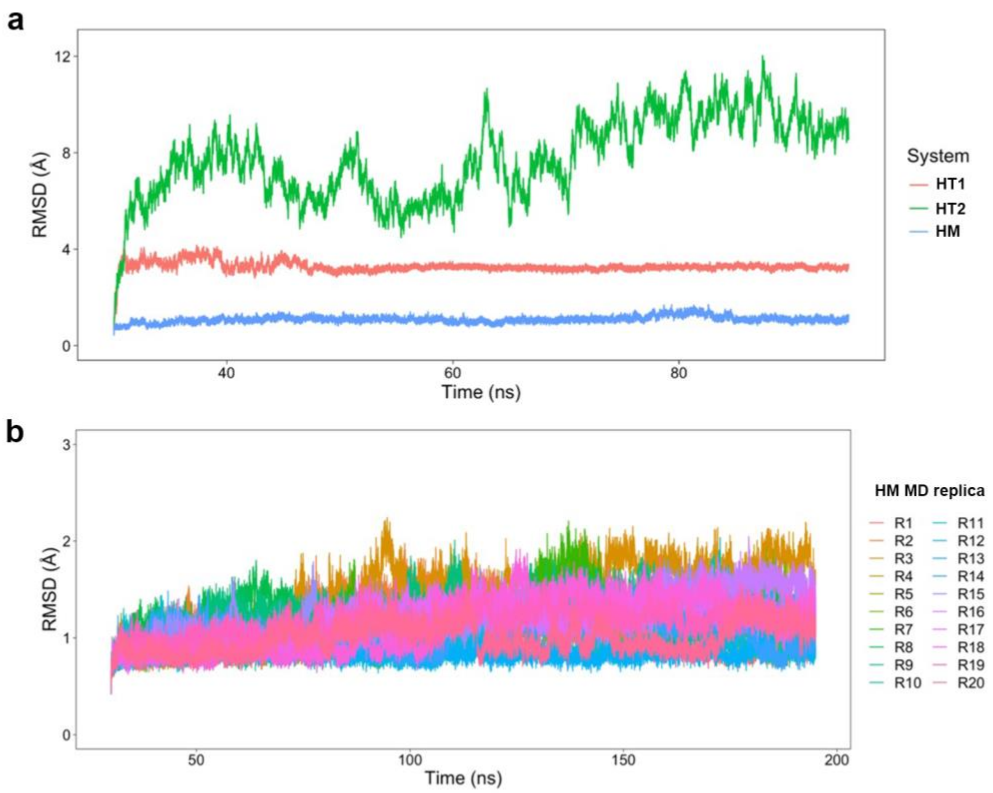

Supplementary Fig. 16 | Stability of Lcl-CTD/C4S during Molecular Dynamics simulations. a, Time evolutions of the RMSD of C_α atoms from the starting structure before energy minimisation for Lcl-CTD in complex with C4S. The HT1 and HT2 plots represent MD simulations starting from different HADDOCK models where one molecule of C4S dp8 was docked against a trimer of Lcl-CTD (3:1 Lcl-CTD:C4S). The HM plot represents a simulation starting from the HADDOCK model used for subsequent analysis. During HADDOCK one molecule of C4S dp8 was docked against a monomer (chain A) of Lcl-CTD (1:1 Lcl-CTD:C4S) and then prior to running the MD simulations it was reconstituted as a trimer (3:1 Lcl-CTD:C4S). **b,** The RMSD values for all the replicas starting from the HM model. For **a** and **b**, RMSD values were calculated after best-fit superimposition of each frame to the reference structure and the highly flexible N-terminal residues (271-277) were not included in the calculation. The initial equilibration stages with positional restraints were omitted from the plot.

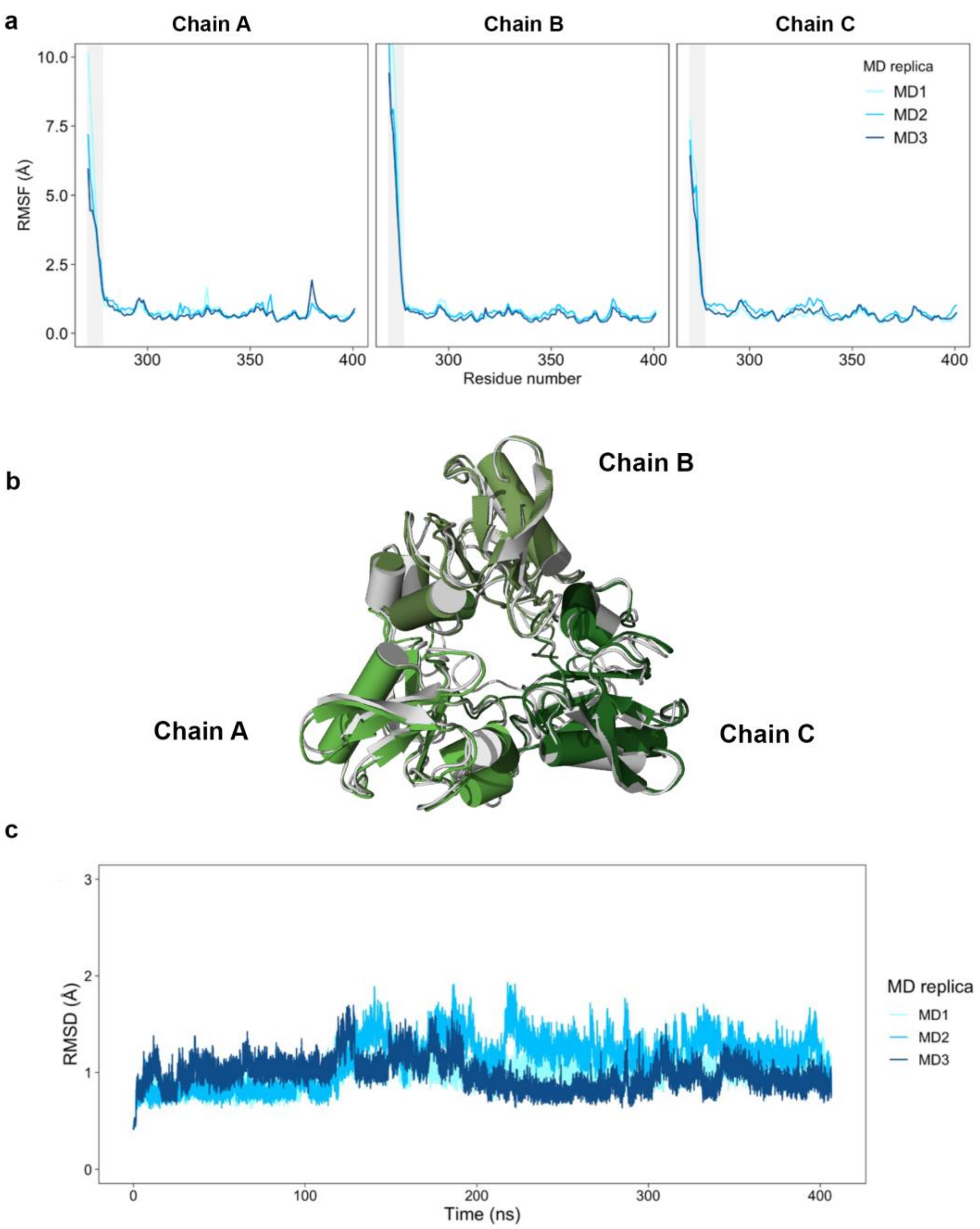

Supplementary Fig. 17 | Molecular Dynamics simulations of Lcl-CTD. a, RMSF profiles for each monomer and each replica of the Lcl-CTD simulations. The grey area highlights the position of the first 7 residues (271 to 277) for each monomer. RMSF values are calculated considering only C_α atoms and on frames saved every 1 ps. **b,** Representative structure of the most populated cluster (white, population=79.7%) from the MD simulations superimposed to the initial crystal structure (green hues). **c,** Time evolution of the RMSD of C_α atoms from the starting structure before energy minimisation for Lcl-CTD alone.

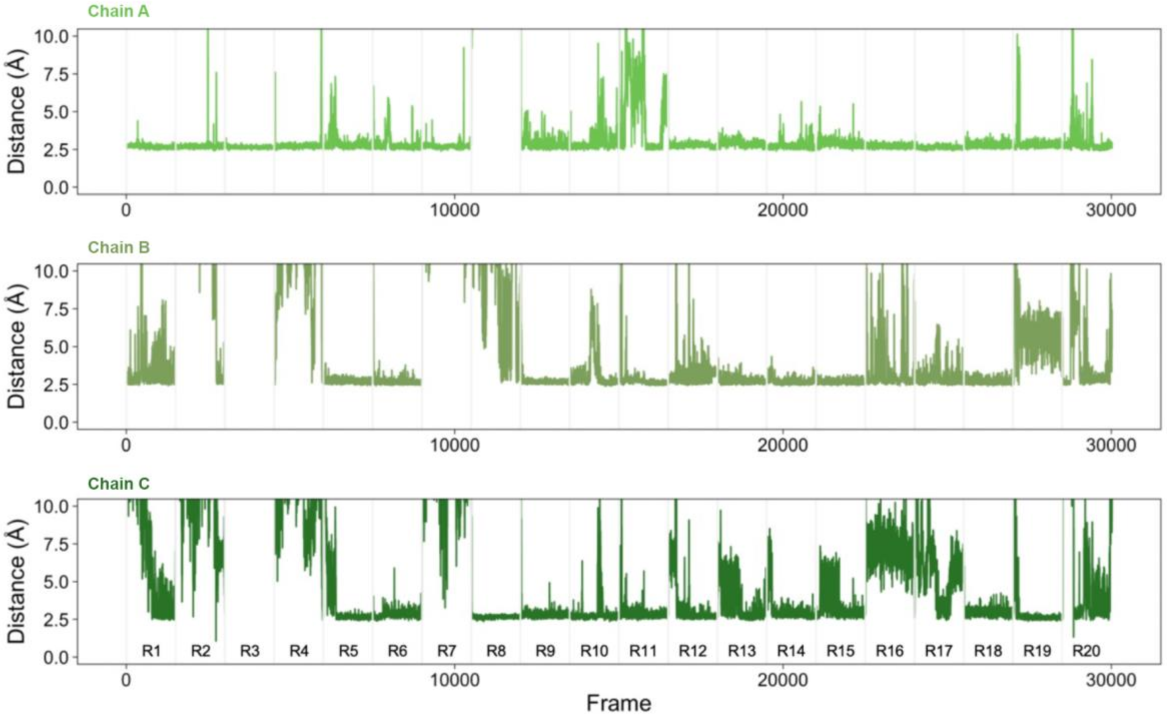

Supplementary Fig. 18 | C4S binding to Lcl-CTD. Time evolution of the distance between C4S and each chain of the Lcl-CTD trimer during the 20 MD replicas (production only). A grey vertical line separates different replicas. The distance was calculated as the minimum distance between all possible pairs of non-hydrogen atoms from C4S and Lcl-CTD. Analysis of the distances shows that C4S can be in contact with a single chain (36.2% of the frames from all replicas), 2 chains (34.8%) or across the whole trimer (27.7%).

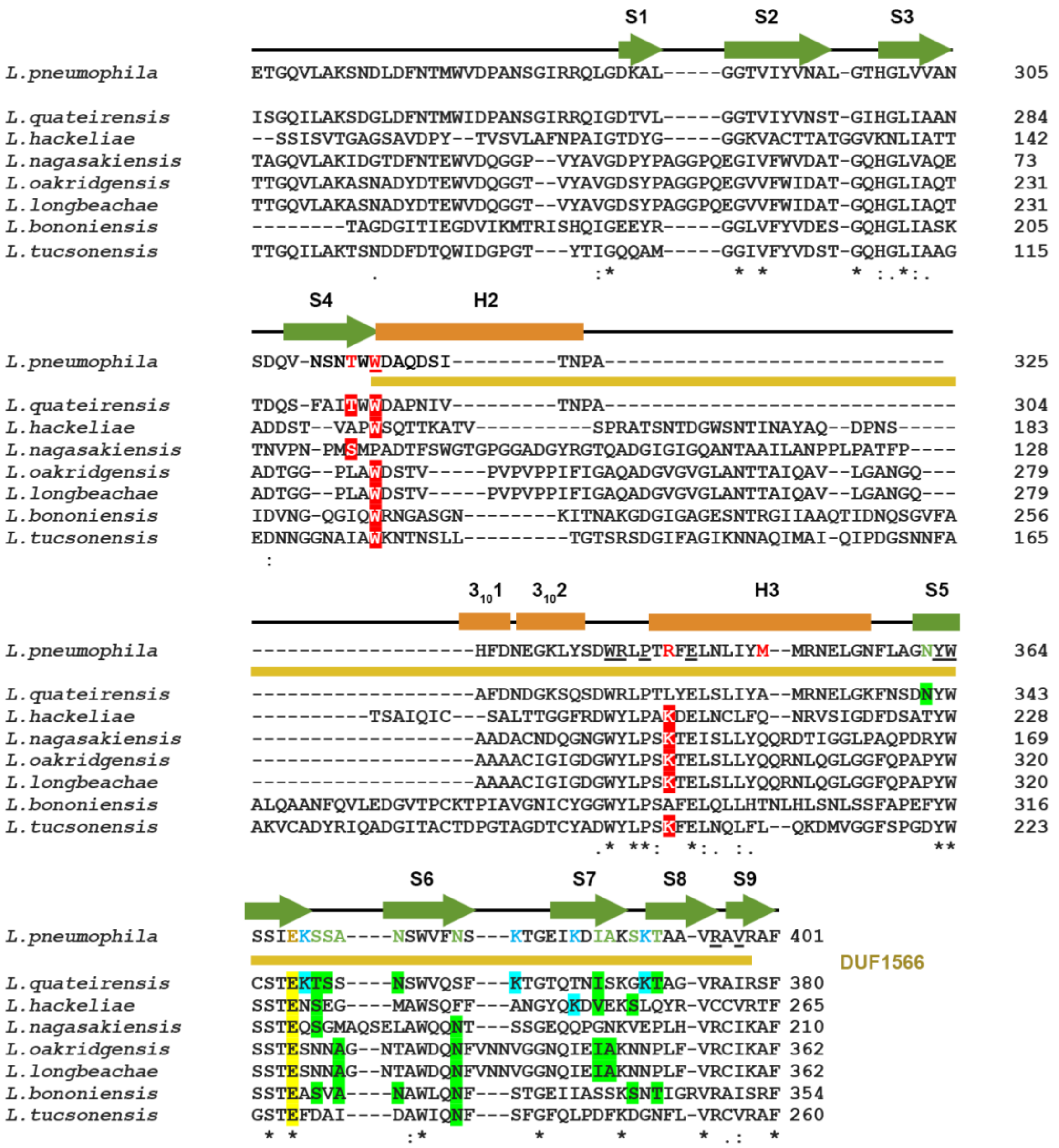

Supplementary Fig. 19 | Sequence alignment of Lcl across the *Legionella* genus. Amino acid positions with 100% identical, >50% identical and similar residues are indicated by asterisk (*), colon (:), and period (.), respectively. Residues in *L. pneumophila* 130b Lcl-CTD that influence GAG binding are coloured red (identified by NMR), green (identified by MD), yellow (internal glutamate; identified by ELISA) and blue (surface lysines; identified by ELISA). Identical residues in other species are shaded in the same colour. Secondary structure elements and the position of DUF1566 are shown for *L. pneumophila* 130b Lcl-CTD, with related residues in DUF1566 that are highly conserved, underlined in the 130b sequence.

Supplementary Table 1 | Domain boundaries of Lcl from *L. pneumophila* 130b strain

The predicted N-terminal amphipathic helix is underlined with residues predicted to form the hydrophobic face in bold[7].

Flexible N-terminus<br>(residues 1 to 30)	KSNPASQAY**V**DGK**V**SE**L**KNE**L**TNK**I**NSIPS
CLR region<br>(residues 31 to 251)	GPQGPRGDKGEAGPKGDRGEAGPQGLPGPKGDRGEAGPQGLPGPKGDRGEAGPQGLPGPKGDRG<br>EAGPQGLPGPKGDRGEAGPQGLPGPQGLPGPKGDKGEAGPQGLPGPKGDKGEAGPQGLPGPKGD<br>KGEAGAVGPQGMPGPKGDKGEAGPQGLPGPKGDRGEAGPQGLPGPKGDRGEAGPQGLPGPKGDK<br>GETGAVGPQGMPGPKGEAGDDGQGVPAGG
C-terminal domain (CTD)<br>(residues 252 to 401)	ETGQVLAKSNDLDFNTMWVDPANSGIRRQLGDKALGGTVIYVNALGTHGLVVANSDQVNSNTWW<br>DAQDSITNPAHFDNEGKLYSDWRLPTRFELNLIYMMRNELGNFLAGNYWSSIEKSSANSWVFNS<br>KTGEIKDIAKSKTAAVRAVRAF

Supplementary Table 2 | X-ray data collection and refinement statistics

**Crystal Parameters**		
Space group	*C*2	*P*321
Cell dimensions (Å; °)	*a*=91.60, *b*=52.87, *c*=97.27; α=γ=90, β=121.09	*a*=74.91, *b*=74.91, *c*=94.97; α=β=90, γ=120
**Data collection**		
Beamline	DLS I03	DLS I03
Wavelength (Å)	0.97969	0.97625
Resolution (Å)	45.56-1.90 (1.95-1.90)	64.87-1.90 (1.93-1.90)
Unique observations	34006 (2460)	24856 (1235)
R_{merge} (%)	0.058 (0.148)	0.074 (0.541)
$\langle I \rangle/\sigma I$	16.7 (6.7)	28.7 (5.8)
Completeness (%)	99.3 (97.3)	100.0 (99.8)
Redundancy	3.3 (2.8)	19.3 (14.8)
Wilson B (Å)	17.3	24.1
**Phasing**		
Figure of merit (accentric/centric)	0.251/0.010	-
Phasing power	0.865	-
**Refinement**		
R_{work}/R_{free} (%)	15.8/18.8	16.0/18.7
Number of protein residues	387	262
Number of water molecules	397	194
Number of ligands	0	2 SO_4^{2}
$\langle B \rangle$ protein	19.1	27.8
$\langle B \rangle$ waters	28.3	35.8
$\langle B \rangle$ ligands	-	34.5
rmsd stereochemistry		
Bond lengths (Å)	0.005	0.008
Bond angles (°)	1.180	1.473
Ramachandran analysis		
Residues in outlier regions (%)	0.0	0.0
Residues in favoured regions (%)	97.7	98.8
Residues in allowed regions (%)	100	100

Numbers in parentheses refer to the outermost resolution shell.
$R_{sym} = \sum|I - \langle I \rangle|/\sum I$ where I is the integrated intensity of a given reflection and $\langle I \rangle$ is the mean intensity of multiple corresponding symmetry-related reflections.
$R_{work} = \sum||F_o| - |F_c||/\sum F_o$ where F_o and F_c are the observed and calculated structure factors, respectively.
$R_{free} = R_{work}$ calculated using 10% random data excluded from the refinement.
rmsd stereochemistry is the deviation from ideal values.
Ramachandran analysis was carried out using Molprobity [8]

Supplementary Table 3 | SAXS data and refinement statistics

**SAXS data collection**	WT	R342A peak 1	R342A peak 2	E368A	K369A	K380A	K385A	D386A	K391A
Beamline	DLS B21	DLS B21	DLS B21	DLS B21	DLS B21	DLS B21	DLS B21	DLS B21	DLS B21
Wavelength (Å)	1.0	1.0	1.0	1.0	1.0	1.0	1.0	1.0	1.0
q Range ($Å^{-1}$)	0.003 to 0.44	0.003 to 0.44	0.003 to 0.44	0.003 to 0.44	0.003 to 0.44	0.003 to 0.44	0.003 to 0.44	0.003 to 0.44	0.003 to 0.44
**Structural parameters**									
I(0)	0.091 ± 5.6e-05	0.091 ± 2.3e-04	0.075 ± 4.5e-05	0.090 ± 5.4e-05	0.091 ± 5.2e-05	0.094 ± 8.2e-05	0.092 ± 1.0e-04	0.093 ± 6.3e-05	0.093 ± 5.2e-05
R_g (nm) (from Guinier)	2.77 ± 0.10	2.68 ± 0.04	1.84 ± 0.15	2.79 ± 0.36	2.78 ± 0.18	2.79 ± 0.04	2.81 ± 0.18	2.83 ± 0.02	2.77 ± 0.01
R_g (nm) (from P(r))	2.85 ± 0.01	2.75 ± 0.01	1.85 ± 0.01	2.82 ± 0.01	2.85 ± 0.01	2.86 ± 0.01	2.88 ± 0.01	2.84 ± 0.01	2.83 ± 0.01
D_{max} (nm) (from P(r))	9.70	9.38	6.44	9.89	9.73	9.77	9.84	9.77	9.49
MW (kDa) (from sequence)	18549	18464	18464	18475	18492	18492	18492	18479	18492
MW (kDa) (from SAXS)	50877	47126	18809	50626	51962	51517	51667	51522	51493

Supplementary Table 4 | SAXS ensemble optimization parameters.

R_{flex} (random) (%)	88.10 (84.55)
R_{sig}	4.40
χ^2	1.05
Cluster 1 (%)	30
Cluster 2 (%)	20
Cluster 3 (%)	50
Final ensemble R_g (nm)	2.82
Final ensemble D_{max} (nm)	9.18

Supplementary Table 5 | Lcl homologs from the DUF1566 gly_rich_SclB superfamily.

Description	Species	NIH accession code
DUF1566 domain-containing protein	Legionella pneumophila	WP_010948344.1
DUF1566 domain-containing protein	Legionella pneumophila	WP_015961725.1
DUF1566 domain-containing protein	Legionella pneumophila	WP_011216516.1
Legionella vir region protein	Flavobacteria. bacterium BAL38	EAZ94970.1
Hypothetical protein GOS_6310005, partial (marine metagenome)	N/A	ECC71828.1
DUF1566 domain-containing protein	Legionella pneumophila	WP_011947561.1
DUF1566 domain-containing protein	Legionella pneumophila	WP_013101911.1
DUF1566 domain-containing protein	Legionella pneumophila	WP_040185895.1
DUF1566 domain-containing protein	Legionella pneumophila	WP_061563548.1
DUF1566 domain-containing protein	Legionella pneumophila	WP_062725249.1
HbP1	Legionella pneumophila	ADN03210.1
DUF1566 domain-containing protein	Legionella pneumophila	WP_014844754.1
DUF1566 domain-containing protein	Legionella pneumophila	WP_015443987.1
DUF1566 domain-containing protein	Legionella pneumophila	WP_016357032.1
DUF1566 domain-containing protein, partial	Legionella pneumophila	WP_021459955.1
DUF1566 domain-containing protein	Legionella oakridgensis	WP_025385719.1
DUF1566 domain-containing protein	Legionella oakridgensis	WP_025385919.1
DUF1566 domain-containing protein	Legionella oakridgensis	WP_035896162.1
DUF1566 domain-containing protein, partial	Legionella pneumophila	WP_027264466.1
DUF1566 domain-containing protein	Legionella pneumophila	WP_027267710.1
DUF1566 domain-containing protein, partial	Legionella pneumophila	WP_032828702.1
DUF1566 domain-containing protein, partial	Legionella pneumophila	WP_032828931.1
DUF1566 domain-containing protein, partial	Legionella pneumophila	WP_032832228.1
DUF1566 domain-containing protein, partial	Legionella pneumophila	WP_032833288.1
DUF1566 domain-containing protein, partial	Legionella pneumophila	WP_038839654.1
DUF1566 domain-containing protein, partial	Legionella pneumophila	WP_040146561.1
DUF1566 domain-containing protein, partial	Legionella pneumophila	WP_040147717.1
DUF1566 domain-containing protein	Legionella pneumophila	WP_040164635.1
DUF1566 domain-containing protein, partial	Legionella pneumophila	WP_040176715.1
DUF1566 domain-containing protein, partial	Legionella pneumophila	WP_040183632.1
DUF1566 domain-containing protein	Legionella pneumophila	WP_040184835.1
DUF1566 domain-containing protein, partial	Legionella pneumophila	WP_040187765.1
DUF1566 domain-containing protein, partial	Legionella pneumophila	WP_040190847.1
DUF1566 domain-containing protein	Legionella pneumophila	WP_042237472.1
DUF1566 domain-containing protein, partial	Legionella pneumophila	WP_042637136.1
DUF1566 domain-containing protein, partial	Legionella pneumophila	WP_042645969.1
DUF1566 domain-containing protein	Legionella pneumophila	WP_042647776.1
DUF1566 domain-containing protein, partial	Legionella pneumophila	WP_042647794.1
DUF1566 domain-containing protein, partial	Legionella pneumophila	WP_042647975.1
DUF1566 domain-containing protein, partial	Legionella pneumophila	WP_042650802.1
DUF1566 domain-containing protein, partial	Legionella pneumophila	WP_042655488.1
DUF1566 domain-containing protein	Legionella pneumophila	WP_042754707.1
DUF1566 domain-containing protein	Legionella pneumophila	WP_059428048.1
DUF1566 domain-containing protein	Legionella pneumophila	WP_059428638.1
DUF1566 domain-containing protein, partial	Legionella pneumophila	WP_044499023.1
DUF1566 domain-containing protein, partial	Legionella pneumophila	WP_044552931.1
DUF1566 domain-containing protein, partial	Legionella pneumophila	WP_047661805.1
DUF1566 domain-containing protein, partial	Legionella pneumophila	WP_059411200.1
DUF1566 domain-containing protein, partial	Legionella pneumophila	WP_059431367.1
DUF1566 domain-containing protein	Legionella pneumophila	WP_060871594.1
DUF1566 domain-containing protein	Legionella pneumophila	WP_061469204.1
DUF1566 domain-containing protein	Legionella pneumophila	WP_061684452.1
DUF1566 domain-containing protein	Legionella pneumophila	WP_061691911.1
DUF1566 domain-containing protein	Legionella pneumophila	WP_061635485.1
DUF1566 domain-containing protein	Legionella pneumophila	WP_061710309.1
DUF1566 domain-containing protein	Legionella pneumophila	WP_061638011.1
DUF1566 domain-containing protein	Legionella pneumophila	WP_061832330.1
DUF1566 domain-containing protein	Legionella pneumophila	WP_061515237.1

DUF1566 domain-containing protein	Legionella pneumophila	WP_230305795.1
DUF1566 domain-containing protein	Legionella pneumophila	WP_061690779.1
DUF1566 domain-containing protein	Legionella pneumophila	WP_061722744.1
DUF1566 domain-containing protein	Legionella pneumophila	WP_061722072.1
DUF1566 domain-containing protein	Legionella pneumophila	WP_061637081.1
DUF1566 domain-containing protein	Legionella pneumophila	WP_061723125.1
DUF1566 domain-containing protein	Legionella pneumophila	WP_061716791.1
DUF1566 domain-containing protein	Legionella pneumophila	WP_061836912.1
DUF1566 domain-containing protein	Legionella pneumophila	WP_061639199.1
DUF1566 domain-containing protein	Legionella pneumophila	WP_061723636.1
DUF1566 domain-containing protein	Legionella pneumophila	WP_061398413.1
DUF1566 domain-containing protein	Legionella pneumophila	WP_061684485.1
DUF1566 domain-containing protein	Legionella pneumophila	WP_061828234.1
DUF1566 domain-containing protein	Legionella pneumophila	WP_061635538.1
DUF1566 domain-containing protein	Legionella pneumophila	WP_061723696.1
DUF1566 domain-containing protein	Legionella pneumophila	WP_061828204.1
DUF1566 domain-containing protein	Legionella pneumophila	WP_061827839.1
DUF1566 domain-containing protein	Legionella pneumophila	WP_061828230.1
DUF1566 domain-containing protein	Legionella pneumophila	WP_061724091.1
DUF1566 domain-containing protein	Legionella pneumophila	WP_061467335.1
DUF1566 domain-containing protein	Legionella pneumophila	WP_061773537.1
DUF1566 domain-containing protein	Legionella pneumophila	WP_061467096.1
DUF1566 domain-containing protein	Legionella pneumophila	WP_061480980.1
DUF1566 domain-containing protein	Legionella pneumophila	WP_061481517.1
DUF1566 domain-containing protein	Legionella pneumophila	WP_061483720.1
DUF1566 domain-containing protein	Legionella pneumophila	WP_061512424.1
DUF1566 domain-containing protein	Legionella pneumophila	WP_061513719.1
DUF1566 domain-containing protein	Legionella pneumophila	WP_061773165.1
DUF1566 domain-containing protein	Legionella pneumophila	WP_061784476.1
DUF1566 domain-containing protein, partial	Legionella pneumophila	WP_062721381.1
DUF1566 domain-containing protein, partial	Legionella pneumophila	WP_062739669.1
DUF1566 domain-containing protein, partial	Legionella pneumophila	WP_062739721.1
DUF1566 domain-containing protein, partial	Legionella pneumophila	WP_062756802.1
DUF1566 domain-containing protein, partial	Legionella pneumophila	WP_062777099.1
DUF1566 domain-containing protein	Legionella pneumophila	WP_062896185.1
DUF1566 domain-containing protein	Legionella oakridgensis	WP_064101346.1
DUF1566 domain-containing protein	Legionella pneumophila	WP_070322485.1
DUF1566 domain-containing protein	Legionella pneumophila	WP_070322308.1
MAG: hypothetical protein A3D92_17110	Bacteroidetes bacterium RIFCSPHIGHO2_02_FULL_44_7	OFZ16950.1
MAG: hypothetical protein A3D31_13075	Candidatus Fluviicola riflensis	OGS77914.1
DUF1566 domain-containing protein, partial	Legionella pneumophila	WP_143460085.1
DUF1566 domain-containing protein, partial	Legionella pneumophila	WP_143460548.1
MAG: hypothetical protein COA38_21500	Fluviicola sp.	PHR16987.1
collagen triple helix repeat protein	Comamonas sp. 26	PIG08876.1
MAG: hypothetical protein CK423_04720	Legionella sp.	PJD94984.1
MAG: hypothetical protein CK426_01620	Legionella sp.	PJD99773.1
DUF1566 domain-containing protein	Nonlabens sp. MB-3u-79	WP_101013415.1
DUF1566 domain-containing protein	Legionella pneumophila	WP_106174748.1
DUF1566 domain-containing protein	Legionella pneumophila	WP_106184284.1
DUF1566 domain-containing protein	Legionella pneumophila	WP_106195271.1
DUF1566 domain-containing protein	Legionella pneumophila	WP_106225763.1
DUF1566 domain-containing protein	Legionella pneumophila	WP_106229744.1
DUF1566 domain-containing protein	Legionella pneumophila	WP_110570296.1
DUF1566 domain-containing protein	Oceanihabitans sediminis	WP_123796521.1
DUF1566 domain-containing protein	Oceanihabitans sediminis	RCU56654.1
DUF1566 domain-containing protein	Legionella pneumophila	WP_114693300.1
DUF1566 domain-containing protein, partial	Legionella pneumophila	WP_114674093.1
tail fiber protein	Legionella longbeachae	STY20446.1
DUF1566 domain-containing protein	Legionella quateirensis	WP_115148663.1
uncharacterized protein DUF1566, partial	Winogradskyella pacifica	REE25412.1
DUF1566 domain-containing protein	Legionella pneumophila	WP_120061973.1
DUF1566 domain-containing protein	Legionella pneumophila	WP_134984243.1
DUF1566 domain-containing protein	Legionella pneumophila	WP_129368547.1

DUF1566 domain-containing protein	Legionella quateirensis	WP_133141067.1
DUF1566 domain-containing protein, partial	Legionella pneumophila	WP_134921750.1
DUF1566 domain-containing protein	Legionella pneumophila	WP_136669451.1
DUF1566 domain-containing protein, partial	Legionella pneumophila	WP_136691115.1
DUF1566 domain-containing protein	Legionella pneumophila	WP_136627821.1
DUF1566 domain-containing protein	Legionella pneumophila	WP_136633845.1
DUF1566 domain-containing protein	Legionella pneumophila	WP_136648505.1
MAG: DUF1566 domain-containing protein, partial	Gammaproteobacteria bacterium	TLZ59698.1
MAG: DUF1566 domain-containing protein	Chitinophagaceae bacterium	TVR83543.1
DUF1566 domain-containing protein, partial	Legionella pneumophila	WP_148654818.1
hypothetical protein EZS27_000721<br>(termite gut metagenome)	N/A	KAA6351924.1
Hypothetical protein	Cupriavidus sp. SK-3	WP_152561645.1
DUF1566 domain-containing protein, partial	Legionella pneumophila	WP_154231773.1
unannotated protein (freshwater metagenome)	N/A	CAB4331133.1
DUF1566 domain-containing protein, partial	Legionella pneumophila	WP_154694957.1
DUF1566 domain-containing protein, partial	Legionella pneumophila	WP_154698949.1
DUF1566 domain-containing protein, partial	Legionella pneumophila	WP_154698961.1
DUF1566 domain-containing protein, partial	Legionella pneumophila	WP_154699083.1
DUF1566 domain-containing protein, partial	Legionella pneumophila	WP_154699070.1
DUF1566 domain-containing protein, partial	Legionella pneumophila	WP_154699188.1
DUF1566 domain-containing protein, partial	Legionella pneumophila	WP_154699211.1
DUF1566 domain-containing protein, partial	Legionella pneumophila	WP_154699232.1
DUF1566 domain-containing protein, partial	Legionella pneumophila	WP_154699253.1
DUF1566 domain-containing protein, partial	Legionella pneumophila	WP_154699450.1
DUF1566 domain-containing protein, partial	Legionella pneumophila	WP_154699477.1
DUF1566 domain-containing protein, partial	Legionella pneumophila	WP_154699519.1
DUF1566 domain-containing protein, partial	Legionella pneumophila	WP_154699508.1
DUF1566 domain-containing protein, partial	Legionella pneumophila	WP_154699564.1
DUF1566 domain-containing protein, partial	Legionella pneumophila	WP_154699554.1
DUF1566 domain-containing protein, partial	Legionella pneumophila	WP_154699579.1
DUF1566 domain-containing protein, partial	Legionella pneumophila	WP_154699623.1
DUF1566 domain-containing protein, partial	Legionella pneumophila	WP_154699798.1
DUF1566 domain-containing protein, partial	Legionella pneumophila	WP_161503312.1
DUF1566 domain-containing protein, partial	Legionella pneumophila	WP_161503617.1
DUF1566 domain-containing protein, partial	Legionella pneumophila	WP_161505509.1
DUF1566 domain-containing protein, partial	Legionella pneumophila	WP_161513085.1
DUF1566 domain-containing protein, partial	Legionella pneumophila	WP_161513358.1
DUF1566 domain-containing protein, partial	Legionella pneumophila	WP_161513925.1
DUF1566 domain-containing protein	Legionella pneumophila	WP_161514298.1
DUF1566 domain-containing protein, partial	Legionella pneumophila	WP_161514748.1
DUF1566 domain-containing protein, partial	Legionella pneumophila	WP_161516834.1
Collagen triple helix repeat<br>(uncultured Caudovirales phage)	N/A	CAB4171944.1
DUF1566 domain-containing protein	Winogradskyella wichelsiae	WP_179375955.1
hypothetical protein GCM10010832_23760	Psychroflexus planctonicus	GGE43047.1
DUF1566 domain-containing protein	Psychroflexus planctonicus	WP_194428404.1
phage tail protein	Legionella pneumophila	WP_281162213.1
DUF1566 domain-containing protein, partial	Legionella pneumophila	WP_247337804.1
DUF1566 domain-containing protein, partial	Legionella pneumophila	WP_247386728.1
DUF1566 domain-containing protein	Legionella pneumophila	WP_241505424.1
DUF1566 domain-containing protein	Legionella pneumophila	WP_241507376.1
DUF1566 domain-containing protein, partial	Legionella pneumophila	WP_247358519.1
hypothetical protein	Comamonas sp. 26	WP_199173708.1
DUF1566 domain-containing protein	Legionella sp. PATHC032	WP_265719455.1
DUF1566 domain-containing protein, partial	Legionella pneumophila	WP_247094671.1
DUF1566 domain-containing protein, partial	Legionella pneumophila	WP_241510043.1
DUF1566 domain-containing protein, partial	Legionella pneumophila	WP_241508403.1
DUF1566 domain-containing protein	Methylomonas paludis	WP_215584844.1
DUF1566 domain-containing protein, partial	Legionella pneumophila	WP_229309814.1
DUF1566 domain-containing protein, partial	Legionella pneumophila	WP_230308248.1
DUF1566 domain-containing protein, partial	Legionella pneumophila	WP_230314272.1
DUF1566 domain-containing protein	Legionella pneumophila	WP_236715301.1
hypothetical protein	Legionella bononiensis	WP_238400292.1

DUF1566 domain-containing protein	Legionella bononiensis	WP_238400409.1
DUF1566 domain-containing protein, partial	Legionella quateirensis	WP_238585567.1
DUF1566 domain-containing protein	Legionella pneumophila	WP_241507749.1
DUF1566 domain-containing protein, partial	Legionella pneumophila	WP_241534195.1
DUF1566 domain-containing protein, partial	Legionella pneumophila	WP_241506022.1
DUF1566 domain-containing protein, partial	Legionella pneumophila	WP_241505578.1
DUF1566 domain-containing protein	Legionella pneumophila	WP_241533060.1
DUF1566 domain-containing protein	Legionella pneumophila	WP_241535769.1
	Legionella pneumophila	WP_241534112.1
DUF1566 domain-containing protein, partial	Legionella pneumophila	WP_241534982.1
DUF1566 domain-containing protein	Legionella pneumophila	WP_241532330.1
DUF1566 domain-containing protein	Thiocystis minor	WP_242523155.1
hypothetical protein	Methylobacter sp. S3L5C	WP_243219470.1
DUF1566 domain-containing protein, partial	Legionella pneumophila	WP_247356958.1
phage tail protein	Legionella pneumophila	WP_269568483.1
DUF1566 domain-containing protein, partial	Legionella pneumophila	WP_269562955.1
hypothetical protein	Winogradskyella pacifica	WP_257018464.1
hypothetical protein	Winogradskyella wichelsiae	WP_262886520.1
hypothetical protein	Bacteroidia bacterium	GHS95945.1
phage tail protein	Legionella pneumophila	WP_265665447.1
phage tail protein	Legionella sp. PATHC039	WP_265732188.1
phage tail protein	Legionella pneumophila	WP_265672815.1
phage tail protein	Legionella pneumophila	WP_265672181.1
phage tail protein	Legionella pneumophila	WP_269568141.1
phage tail protein, partial	Legionella pneumophila	WP_269563856.1
DUF1566 domain-containing protein, partial	Legionella pneumophila	WP_269568104.1
phage tail protein	Legionella pneumophila	WP_269568937.1
DUF1566 domain-containing protein, partial	Legionella pneumophila	WP_269567031.1
phage tail protein	Legionella pneumophila	WP_269564737.1
phage tail protein	Legionella pneumophila	WP_269564955.1
DUF1566 domain-containing protein, partial	Legionella pneumophila	WP_269565042.1
phage tail protein, partial	Legionella pneumophila	WP_269566652.1
DUF1566 domain-containing protein, partial	Legionella pneumophila	WP_269566086.1
DUF1566 domain-containing protein, partial	Legionella pneumophila	WP_269569356.1
DUF1566 domain-containing protein, partial	Legionella pneumophila	WP_269562660.1
DUF1566 domain-containing protein, partial	Legionella pneumophila	WP_269568473.1
phage tail protein	Legionella pneumophila	WP_269568720.1
DUF1566 domain-containing protein, partial	Legionella pneumophila	WP_272838090.1
DUF1566 domain-containing protein, partial	Legionella pneumophila	WP_272850246.1
MAG: Uncharacterised protein	Formosa sp. Hel1_33_131	CAI8189797.1
phage tail protein	Legionella pneumophila	WP_281489849.1
phage tail protein	Legionella pneumophila	WP_283324708.1

Supplementary Table 6 | Average frequency of occurrence of Lcl-CTD/C4S contacts during MD simulations.

Residue[a]	Chain A[b] (%)	Chain B[b] (%)	Chain C[b] (%)
S371	40.9	40.3	37.6
A372	29.2	31.1	25.7
**K391**	32.4	24.8	19.3
S390	20.9	36.5	17.2
A388	21.9	22.6	18.2
S370	26.9	14.7	20.7
N373	24.4	11.6	14.5
W315	11.1	22.3	9.6
I387	14.8	4.2	7.1
**K385**	16.3	1.8	4.8
**K369**	13.4	3.1	3.0
T392	10.1	0.8	7.5
N362	13.4	0.4	3.7
N378	10.6	0.3	0.3
**K380**	10.2	0.2	0.1

[a]Residues are sorted by decreasing average frequency. Lysines are highlighted in bold.
[b]The frequency was calculated as average over all the replicas (production only). Only residues with a frequency ≥ 10% for at least one chain are reported.

Supplementary Table 7 | Average frequency of occurrence of Lcl-CTD/C4S hydrogen bonds during MD simulations.

Residue[a]	Chain A[b] (%)	Chain B[b] (%)	Chain C[b] (%)
S371	39.7	36.8	40.8
S390	11.7	27.8	10.1
**K391**	19.1	11.7	9.7
T392	15.9	1.9	11.8
N373	10.6	7.8	8.4
S370	12.0	4.2	4.9
N362	14.6	0.7	4.6
**K385**	11.8	0.6	2.9

[a]Residues are sorted by decreasing average frequency. Lysines are highlighted in bold. Hydrogen bonding interactions were detected using Visual Molecular Dynamics[9] with a Donor-Acceptor distance threshold of 3.5 Å and a Hydrogen-Donor-Acceptor angle of 30°.
[b]The frequency was calculated as average over all the replicas (production only). When more than one donor/acceptor per residue was involved in hydrogen bonding, the frequency values were added together. Only residues with a frequency >= 10% for at least one chain are reported.

Supplementary Table 8 | Primers, plasmids and strains used in this study

Primer	Description	Sequence (5' to 3')
lcl-UpF	Gene KO – *L. pneumophila* 130b *lcl* upstream F	ATACGTACACGAAGGCGACC
lcl -UpR	Gene KO – *L. pneumophila* 130b *lcl* upstream R	TCTTTATTTCCCACTATATGCTCACTTG
lcl-DownF	Gene KO – *L. pneumophila* 130b *lcl* downstream F	GTATGGCAAAACTTCAATTTTGCT
lcl -DownR	Gene KO – *L. pneumophila* 130b *lcl* downstream R	AGAAGTACGCCCTGGTTTCG
lcl-P1	Gene KO - Kn-resistance cassette F	AAATAAAAGAGACAAGTGAGCATATAGTGG GAAATAAAGAGTGTAGGCTGGAGCTGCTTC
lcl-P2	Gene KO - Kn-resistance cassette R	GAACATTTTCATCCTTGAGCAAAATTGAAGT TTTGCCATACCATATGAATATCCTCCTTA
RLC1	Recombinant expression of *L. pneumophila* 130b Lcl F	GACGACGACAAGATGAAAAGCAATCCGGCC TCGC
RLC2	Recombinant expression of *L. pneumophila* 130b Lcl R	GAGGAGAAGCCCGGttaAAAGGCTCTTACAGC ACGTAC
RLC3	Recombinant expression of *L. pneumophila* 130b Lcl-CTD F	GACGACGACAAGATGGAAACGGGACAAGTC CTTGC
RLC4	Recombinant expression of *L. pneumophila* 130b Lcl-CTD R	GAGGAGAAGCCCGGttaAAAGGCTCTTACAGC ACGTAC

Plasmid	Description	Reference
pET46 Ek/LIC	Expression vector	Novagen
pRLC1	pET46 Ek/LIC expression of *L. pneumophila* Lcl residues 1-401	This study
pRLC2	pET46 Ek/LIC expression of *L. pneumophila* Lcl CTD residues 252-401	This study
pET28b	Expression vector	Novagen
pRLCm1	pET28b expression of *L. pneumophila* Lcl (CTD) residues 252-401 containing R342A mutation	This study
pRLCm2	pET28b expression of *L. pneumophila* Lcl (CTD)residues 252-401 containing E368A mutation	This study
pRLCm3	pET28b expression of *L. pneumophila* Lcl (CTD)residues 252-401 containing K369A mutation	This study
pRLCm4	pET28b expression of *L. pneumophila* Lcl (CTD)residues 252-401 containing K380A mutation	This study
pRLCm5	pET28b expression of *L. pneumophila* Lcl (CTD)residues 252-401 containing K385A mutation	This study
pRLCm6	pET28b expression of *L. pneumophila* Lcl (CTD)residues 252-401 containing D386A mutation	This study
pRLCm7	pET28b expression of *L. pneumophila* Lcl (CTD)residues 252-401 containing K391A mutation	This study

Bacterial strain	Serogroup/ genotype	Reference
L. pneumophila 130b	ATCC strain BAA-74. Served as the wild type and parent for all mutants.	[10,11]
L. pneumophila NU468	*130b strain carrying lcl mutation*	This study
L. pneumophila NU469	*130b strain carrying lcl mutation*	This study
L. pneumophila NU275	*130b strain carrying lspF (T2SS) mutation*	[12]
E. coli DH5a	*F- Φ80lacZΔM15 Δ(lacZYA-argF) U169 recA1 endA1 hsdR17(rk-, mk+) phoA supE44 thi-1 gyrA96 relA1 λ-*	Invitrogen

E. coli BL21 (DE3)	*fhuA2 [lon] ompT gal (λ DE3) [dcm] ΔhsdS*<br>*λ DE3 = λ sBamHIo ΔEcoRI-B int::(lacI::PlacUV5::T7 gene1) i21 Δnin5*	NEB

Supplementary Table 9 | Synthetic genes

Gene	Description	Sequence (5' to 3')
gRLCm1	Lcl-CTD R342A mutant	CCATGGTCCATCATCATCATCATCATGTGGACGACGACGACAAGATGGAAACGGGACAA<br>GTCCTTGCCAAATCAAATGACCTCGATTTCAATACCATGTGGGTTGACCCGGCAAATTC<br>CGGCATTAGGCGGCAATTAGGCGATAAAGCTCTTGGCGGTACAGTGATTTATGTTAATG<br>CGCTAGGTACACACGGGCTTGTAGTAGCAAATTCAGATCAAGTTAATTCAAACACATGG<br>TGGGATGCTCAAGATTCCATAACAAATCCTGCCCACTTTGACAATGAAGGTAAATTATA<br>TTCTGACTGGAGGCTCCCTACCGCGTTTGAATTGAATTTAATTTATATGATGCGAAATG<br>AGCTGGGAAATTTTTTGGCAGGTAATTACTGGAGTTCGATTGAAAAATCATCGGCAAAC<br>AGCTGGGTATTTAATTCTAAAACAGGGGAAATTAAAGACATTGCCAAAAGTAAAACGGC<br>TGCTGTACGTGCTGTAAGAGCCTTTTAACTCGAG
gRLCm2	Lcl-CTD E368A mutant	CCATGGTCCATCATCATCATCATCATGTGGACGACGACGACAAGATGGAAACGGGACAA<br>GTCCTTGCCAAATCAAATGACCTCGATTTCAATACCATGTGGGTTGACCCGGCAAATTC<br>CGGCATTAGGCGGCAATTAGGCGATAAAGCTCTTGGCGGTACAGTGATTTATGTTAATG<br>CGCTAGGTACACACGGGCTTGTAGTAGCAAATTCAGATCAAGTTAATTCAAACACATGG<br>TGGGATGCTCAAGATTCCATAACAAATCCTGCCCACTTTGACAATGAAGGTAAATTATA<br>TTCTGACTGGAGGCTCCCTACCCGTTTTGAATTGAATTTAATTTATATGATGCGAAATG<br>AGCTGGGAAATTTTTTGGCAGGTAATTACTGGAGTTCGATTGCGAAATCATCGGCAAAC<br>AGCTGGGTATTTAATTCTAAAACAGGGGAAATTAAAGACATTGCCAAAAGTAAAACGGC<br>TGCTGTACGTGCTGTAAGAGCCTTTTAACTCGAG
gRLCm3	Lcl-CTD K369A mutant	CCATGGTCCATCATCATCATCATCATGTGGACGACGACGACAAGATGGAAACGGGACAA<br>GTCCTTGCCAAATCAAATGACCTCGATTTCAATACCATGTGGGTTGACCCGGCAAATTC<br>CGGCATTAGGCGGCAATTAGGCGATAAAGCTCTTGGCGGTACAGTGATTTATGTTAATG<br>CGCTAGGTACACACGGGCTTGTAGTAGCAAATTCAGATCAAGTTAATTCAAACACATGG<br>TGGGATGCTCAAGATTCCATAACAAATCCTGCCCACTTTGACAATGAAGGTAAATTATA<br>TTCTGACTGGAGGCTCCCTACCCGTTTTGAATTGAATTTAATTTATATGATGCGAAATG<br>AGCTGGGAAATTTTTTGGCAGGTAATTACTGGAGTTCGATTGAAGCGTCATCGGCAAAC<br>AGCTGGGTATTTAATTCTAAAACAGGGGAAATTAAAGACATTGCCAAAAGTAAAACGGC<br>TGCTGTACGTGCTGTAAGAGCCTTTTAACTCGAG
gRLCm4	Lcl-CTD K380A mutant	CCATGGTCCATCATCATCATCATCATGTGGACGACGACGACAAGATGGAAACGGGACAA<br>GTCCTTGCCAAATCAAATGACCTCGATTTCAATACCATGTGGGTTGACCCGGCAAATTC<br>CGGCATTAGGCGGCAATTAGGCGATAAAGCTCTTGGCGGTACAGTGATTTATGTTAATG<br>CGCTAGGTACACACGGGCTTGTAGTAGCAAATTCAGATCAAGTTAATTCAAACACATGG<br>TGGGATGCTCAAGATTCCATAACAAATCCTGCCCACTTTGACAATGAAGGTAAATTATA<br>TTCTGACTGGAGGCTCCCTACCCGTTTTGAATTGAATTTAATTTATATGATGCGAAATG<br>AGCTGGGAAATTTTTTGGCAGGTAATTACTGGAGTTCGATTGAAAAATCATCGGCAAAC<br>AGCTGGGTATTTAATTCTGCGACAGGGGAAATTAAAGACATTGCCAAAAGTAAAACGGC<br>TGCTGTACGTGCTGTAAGAGCCTTTTAACTCGAG
gRLCm5	Lcl-CTD K385A mutant	CCATGGTCCATCATCATCATCATCATGTGGACGACGACGACAAGATGGAAACGGGACAA<br>GTCCTTGCCAAATCAAATGACCTCGATTTCAATACCATGTGGGTTGACCCGGCAAATTC<br>CGGCATTAGGCGGCAATTAGGCGATAAAGCTCTTGGCGGTACAGTGATTTATGTTAATG<br>CGCTAGGTACACACGGGCTTGTAGTAGCAAATTCAGATCAAGTTAATTCAAACACATGG<br>TGGGATGCTCAAGATTCCATAACAAATCCTGCCCACTTTGACAATGAAGGTAAATTATA<br>TTCTGACTGGAGGCTCCCTACCCGTTTTGAATTGAATTTAATTTATATGATGCGAAATG<br>AGCTGGGAAATTTTTTGGCAGGTAATTACTGGAGTTCGATTGAAAAATCATCGGCAAAC<br>AGCTGGGTATTTAATTCTAAAACAGGGGAAATTGCGGACATTGCCAAAAGTAAAACGGC<br>TGCTGTACGTGCTGTAAGAGCCTTTTAACTCGAG
gRLCm6	Lcl-CTD D386A mutant	CCATGGTCCATCATCATCATCATCATGTGGACGACGACGACAAGATGGAAACGGGACAA<br>GTCCTTGCCAAATCAAATGACCTCGATTTCAATACCATGTGGGTTGACCCGGCAAATTC<br>CGGCATTAGGCGGCAATTAGGCGATAAAGCTCTTGGCGGTACAGTGATTTATGTTAATG<br>CGCTAGGTACACACGGGCTTGTAGTAGCAAATTCAGATCAAGTTAATTCAAACACATGG<br>TGGGATGCTCAAGATTCCATAACAAATCCTGCCCACTTTGACAATGAAGGTAAATTATA<br>TTCTGACTGGAGGCTCCCTACCCGTTTTGAATTGAATTTAATTTATATGATGCGAAATG<br>AGCTGGGAAATTTTTTGGCAGGTAATTACTGGAGTTCGATTGAAAAATCATCGGCAAAC

		AGCTGGGTATTTAATTCTAAAACAGGGGAAATTAAAGCGATTGCCAAAAGTAAAACGGC<br>TGCTGTACGTGCTGTAAGAGCCTTTTAACTCGAG
gRLCm7	Lcl-CTD K391A mutant	CCATGGTCCATCATCATCATCATCATGTGGACGACGACGACAAGATGGAAACGGGACAA<br>GTCCTTGCCAAATCAAATGACCTCGATTTCAATACCATGTGGGTTGACCCGGCAAATTC<br>CGGCATTAGGCGGCAATTAGGCGATAAAGCTCTTGGCGGTACAGTGATTTATGTTAATG<br>CGCTAGGTACACACGGGCTTGTAGTAGCAAATTCAGATCAAGTTAATTCAAACACATGG<br>TGGGATGCTCAAGATTCCATAACAAATCCTGCCCACTTTGACAATGAAGGTAAATTATA<br>TTCTGACTGGAGGCTCCCTACCCGTTTTGAATTGAATTTAATTTATATGATGCGAAATG<br>AGCTGGGAAATTTTTTGGCAGGTAATTACTGGAGTTCGATTGAAAAATCATCGGCAAAC<br>AGCTGGGTATTTAATTCTAAAACAGGGGAAATTAAAGACATTGCCAAAAGTGCGACGGC<br>TGCTGTACGTGCTGTAAGAGCCTTTTAACTCGAG

*Restriction sites are underlined

References

1 Camilloni, C., De Simone, A., Vranken, W. F. & Vendruscolo, M. Determination of secondary structure populations in disordered states of proteins using nuclear magnetic resonance chemical shifts. *Biochemistry* **51**, 2224-2231, doi:10.1021/bi3001825 (2012).

2 Holm, L. & Sander, C. Dali: a network tool for protein structure comparison. *Trends Biochem Sci* **20**, 478-480, doi:10.1016/s0968-0004(00)89105-7 (1995).

3 Huang, K. F. *et al.* Crystal structure of a platelet-agglutinating factor isolated from the venom of Taiwan habu (Trimeresurus mucrosquamatus). *Biochem J* **378**, 399-407, doi:10.1042/BJ20031507 (2004).

4 Horii, K., Okuda, D., Morita, T. & Mizuno, H. Crystal structure of EMS16 in complex with the integrin alpha2-I domain. *J Mol Biol* **341**, 519-527, doi:10.1016/j.jmb.2004.06.036 (2004).

5 Luo, Y. *et al.* Crystal structure of enteropathogenic Escherichia coli intimin-receptor complex. *Nature* **405**, 1073-1077, doi:10.1038/35016618 (2000).

6 Hamburger, Z. A., Brown, M. S., Isberg, R. R. & Bjorkman, P. J. Crystal structure of invasin: a bacterial integrin-binding protein. *Science* **286**, 291-295 (1999).

7 Gautier, R., Douguet, D., Antonny, B. & Drin, G. HELIQUEST: a web server to screen sequences with specific alpha-helical properties. *Bioinformatics* **24**, 2101-2102, doi:10.1093/bioinformatics/btn392 (2008).

8 Davis, I. W., Murray, L. W., Richardson, J. S. & Richardson, D. C. MOLPROBITY: structure validation and all-atom contact analysis for nucleic acids and their complexes. *Nucleic Acids Res* **32**, W615-619, doi:10.1093/nar/gkh398 (2004).

9 Humphrey, W., Dalke, A. & Schulten, K. VMD: visual molecular dynamics. *J Mol Graph* **14**, 33-38, 27-38, doi:10.1016/0263-7855(96)00018-5 (1996).

10 DebRoy, S., Dao, J., Soderberg, M., Rossier, O. & Cianciotto, N. P. Legionella pneumophila type II secretome reveals unique exoproteins and a chitinase that promotes bacterial persistence in the lung. *Proc Natl Acad Sci U S A* **103**, 19146-19151, doi:10.1073/pnas.0608279103 (2006).

11 Schroeder, G. N. *et al.* Legionella pneumophila strain 130b possesses a unique combination of type IV secretion systems and novel Dot/Icm secretion system effector proteins. *J Bacteriol* **192**, 6001-6016, doi:10.1128/JB.00778-10 (2010).

12 Rossier, O., Starkenburg, S. R. & Cianciotto, N. P. Legionella pneumophila type II protein secretion promotes virulence in the A/J mouse model of Legionnaires' disease pneumonia. *Infect Immun* **72**, 310-321, doi:10.1128/IAI.72.1.310-321.2004 (2004).
